# Exposure assessment of the consumers living in Mount Lebanon directorate to antibiotics through medication and red meat intake: A cross-sectional study

**DOI:** 10.14202/vetworld.2019.1395-1407

**Published:** 2019-09

**Authors:** Christelle Bou-Mitri, Paula Hage Boutros, Joelle Makhlouf, Maya Abou Jaoudeh, Najwa El Gerges, Jessy El Hayek Fares, Elie Bou Yazbeck, Hussein Hassan

**Affiliations:** 1 Department of Nursing and Health Sciences, Notre Dame University – Louaize, Zouk Mosbeh, Lebanon; 2 Ministry of Economy and Trade, Beirut, Lebanon; 3 Department of Natural Sciences, School of Arts and Sciences, Lebanese American University, Beirut, Lebanon

**Keywords:** antibiotic knowledge, exposure, meat, penicillin, practices, risk assessment

## Abstract

**Background and Aim:**

The misuse and abuse of antibiotics by human and in animal production are leading to serious threats to global health. This study aimed to assess the dietary exposure of Lebanese consumers to antibiotic residues from the consumption of meat and compare it to that from medication intake.

**Materials and Methods:**

Beef samples (n=61) were collected and analyzed for penicillin residues using enzyme-linked immunosorbent assay and high-performance liquid chromatography. A cross-sectional study recruited 500 participants living in Mount Lebanon, using an interviewer-based questionnaire. The dietary exposure assessment was calculated following the tiered assessment approach.

**Results:**

The results showed that only 44% of the participants reported using antibiotic on doctor’s prescription. Participants with good antibiotic knowledge (6-7/7) are significantly less likely to change antibiotic during treatment and would better use them as compared to those with lower knowledge (p=0.000). Those with lower education and monthly household income are more likely to improperly use antibiotics as compared to those with higher education and income. Penicillin -containing antibiotics were among the most used medications. On the other hand, the prevalence of penicillin residues in the meat samples was 21.3%, though none was above the maximum residue level. The dietary exposure to penicillin through meat consumption was equivalent to 88.3%, 31.9%, and 5.7% of the acceptable daily intake, using Tier 1, 2, and 3 approaches, respectively. Males, single, obese participants, and those with household income below 999,000 Lebanese pound were significantly more exposed to penicillin as compared to other participants due to their high meat consumption.

**Conclusion:**

These findings will provide insight into designing future targeted awareness interventions and adapted policies as efforts toward improving rational use and intake of antibiotics for preventing the development of antibiotic resistance.

## Introduction

Antibiotics are one of the most important medical discoveries. They are naturally occurring semi-synthetic and synthetic compounds that inhibit the growth of bacteria or kill them [1]. They can be administered orally, parenterally, or topically in human and veterinary medicine to treat and prevent communicable diseases as well as for other purposes including growth promotion in food animals [2,3]. Nowadays, the exposure to those drugs whether from their clinical use or intake of animal food products containing residues has increased, especially due to the extensive and uncontrolled use of those agents [4]. The increased prevalence of antibiotic residues in edible food animals is jeopardizing worldwide trade and increasing public health hazards [5-7]. Moreover, the extensive use and sometimes misuse of antibiotics are associated with various harmful effects on the human body [8,9]. It has been reported that short- or long-term exposure to low concentration of antibiotics exposure can lead to the development of allergic reactions, disturbance of natural intestinal microflora, obesity [10-12], type 2 diabetes [13], increased carcinogenicity, teratogenicity and liver poisoning [5,6], multidrug resistance, and increased prevalence of antibiotic resistance [1]. Increasing antibiotic resistance is considered one of the most serious threats to human health by both the US Centers for Disease Control and Prevention and the World Health Organization [14-16]. Recent US estimates indicate that antibiotic-resistant infections afflict more than 2 million people and kill at least 23,000 people annually [14]. It is forecasted that antibiotic resistance will lead to 300 million premature deaths by 2050 if no action is taken which will exceed the predicted combined mortality of both cancer and diabetes [17]. It has been also shown that the treatment of diseases caused by antibiotic-resistant bacteria can lead to increased mortality, morbidity, higher expenses, and prolonged hospital stay [18].

Several factors could contribute to antibiotic resistance including poor prescribing practices by doctors and pharmacists, antibiotic intake without prescriptions, poor adherence to prescription doses, overuse of broad-spectrum antibiotics, and utilization of antibiotics for common acute viral respiratory tract infections [18,19]. Other factors include the lack of policies for restricting and auditing antibiotic prescriptions, the misuse of antibiotics in animals, and lack of guidelines for the use of antibiotics in the livestock production [20,21]. All these factors increase the need for investigating and tackling such practices.

The overall exposure of humans to antibiotics from various sources is a better indication of exploring their toxicological effects. In Lebanon, studies have reported high prevalence of inappropriate antibiotic prescriptions, with inappropriate antibiotic choice, and inaccurate prescribed dose and duration of the treatment [22]. Antibiotics were dispensed in pharmacies without medical prescriptions with higher frequency in lower socioeconomic areas [23]. Moreover, studies have reported that the Lebanese are excessively consuming antibiotics and are also using them without prescription for diseases that do not require them [24,25]. Self-medication was associated with a person’s educational level and knowledge of antibiotics [25]. Recently, studies reported high prevalence of resistant bacteria in the Lebanese community, especially to penicillin [26]. On the other hand, an increase in red meat consumption which could contribute to the increased exposure to antibiotic and other veterinary drugs was reported [27].

To the best of our knowledge, there has been a lack of comprehensive data concerning Lebanese exposure to antibiotic from clinical use and residues in foods. Only one study revealed that penicillin and tetracycline residues in dairy products marketed in Lebanon were below the maximum residue limits (MRLs) [28].

Therefore, the overall objective of this study was to evaluate the exposure of the Lebanese population living in Mount Lebanon to antibiotics through medication use and red meat intake. It aimed to assess the level of knowledge, attitudes, and practices regarding antibiotic usage in a sample of the Lebanese population and determine the sociodemographic characteristics associated with the highest exposure to those drugs. It also aimed to estimate the risk, assess the consumer exposure, and determine the prevalence of antibiotic contamination in red meat and meat products.

## Materials and Methods

### Ethical approval

Ethical approval was not needed for this study.

### Informed consent

Informed consent was obtained from each participant.

### Sampling

A cross-sectional study was conducted to assess the red meat consumption of the Lebanese population, in the Mount Lebanon region. Mount Lebanon was chosen because most of the large supermarkets are located in this region. Data were collected for a period of 1 month extending from November 2016 to December 2016. The sample population included 500 participants restricted to adults. Participants were selected by simple random sampling and were recruited from a total of 11 supermarkets distributed among the six districts of Mount Lebanon relative to their population density. According to the Office for the Coordination of Humanitarian Affairs in Lebanon, the population density was reported in 2014 as follows: 520,165 in Baabda, 428,166 in Metn, 163,872 in Aley, 161,292 in Keserouan, 153,324 in Chouf, and 80,754 in Jbeil with a total of 1,508,658 in Mount Lebanon. Hence, 34.5% of participants (n=173) were recruited from three supermarkets in Baabda, 28.4% (n=142) from three supermarkets in Metn, 10.8% (n=54) from one supermarket in Aley, 10.6% (n=53) from two supermarkets in Keserouan, 10.1% (n=51) from one supermarket in Chouf, and 5.3% (n=27) from one supermarket in Jbeil. Each point of sale was visited once during weekdays and once during weekends, and the shifts for data collection were divided into morning and afternoon to minimize bias and included the most representative sample of the Lebanese population. To calculate the body mass index (BMI), weights of the participants were recorded at the supermarket using a professional calibrated scale Omron Seca (Hamburg, Germany) and height was self-reported. BMI is a simple index of weight-for-height that is commonly used to classify underweight, normal weight, overweight, and obesity in adults. It is defined as the weight in kg over the square of the height in meters (kg/m^2^). People with a BMI <18.5 kg/m^2^ are considered underweight, between 18.5 and 25 kg/m^2^ normal weight, 25-30 kg/m^2^ overweight, and above 30 kg/m^2^ obese.

### Inclusion and exclusion criteria

The recruited participants were 18 years and above. All vegetarians and vegans were excluded from the study. The study was not conducted in a fasting period. All participants were living in the designated area of the study.

### Questionnaire

The questionnaire comprised four major sections assessing (A) sociodemographic characteristics, (B) antibiotic knowledge and attitudes, (C) antibiotic usage, and (D) food frequency questionnaire (FFQ) on 42 of red meat and meat product items. The questionnaire was prepared in English, then translated to Arabic and back translated to English. The participant had the choice of answering the questionnaire in Arabic or English. A pilot study was conducted to get a preliminary validation of the questionnaire (n=20 adults). The clarity, suitability of wording, and the average time needed for its completion were assessed. Then, modifications were made and the results of the pilot study were not included in the data analyses. Each questionnaire required up to 20 min for completion.

### Antibiotic knowledge and trends in antibiotic intake

The knowledge and attitudes toward antibiotic consumption of the Lebanese population and the effect of antibiotic knowledge of antibiotic consumption were assessed. Using the method conducted by Mouhieddine *et al*. [24], a score was calculated to describe the antibiotic knowledge of participants according to the number of correctly answered questions and was categorized as poor, moderate, and good. The score was calculated by providing one point for every correct question answered and no points if the answer was wrong, to reach a maximum of 7 points. The scores were categorized into poor (0-2/7), moderate (3-5/7), or good (6-7/7).

### Red meat and meat product consumption

Data on red meat consumption were collected through a semi-quantitative FFQ that was developed based on the dietary intake of the past 3 months. After a thorough review of the literature and based on the most commonly consumed red meats in Lebanon, the FFQ was established. It included five categories of commonly consumed red meat products in Lebanon that constituted a total of 42 red meat items derived from beef, lamb, and goat.

### Meat sampling

A total of 61 beef meat samples were collected randomly from Mount Lebanon regions between February 2017 and March 2017. The samples comprised steak meat (n=25), minced meat (n=23), liver (n=9), and kidney (n=4). They were collected from butchers (n=17) and supermarkets (n=8) located in Keserwan, Baabda, Metn, and Jbeil. The samples were transferred in a cooler and stored at −20°C until further analyses.

### Enzyme-linked immunosorbent assay (ELISA) procedure

RIDASCREEN^®^ Penicillin ELISA kit (R 2921) was used to quantitatively analyze penicillin residues in the collected meat samples. From the 61 beef meat samples, 20 were chosen randomly and run in duplicates. Seven ampicillin standards (control, 0.125, 0.25, 0.5, 1, 2, and 4 ng/ml) were serially diluted in distilled water, pipetted into the wells, and run in duplicates. The preparation of the reagents, samples, buffer solution, standards, and test procedure was conducted according to the provided manufacturer’s instructional guidelines. The measurement was made photometrically at 450 nm using Thermo 27 Scientific Multiskan Go, and the absorption is inversely proportional to the ampicillin concentration in the sample. The limit of detection (LOD) of the kit is set to be 5.00 μg/kg.

### High-performance liquid chromatography (HPLC) procedure

Agilent 1260 Infinity HPLC with diode array detector was used to verify the positive samples detected by the ELISA procedure. A solid-phase extraction method was used to determine total penicillin residues in the positive samples. The HPLC analysis was conducted according to the United States Department of Agriculture Food Safety and Infection Service, National Residue Program to detect residues in meat, poultry, and eggs described by Barton, Ball, Huber, and Onigbinde [29-31], with few variations in mobile phase and sample preparation described above. A reversed-phase column Agilent Eclipse Plus C18 (3.5 μM, 100 mm×4.6 mm) was used to quantify total penicillin residues in the positive samples under the following conditions: Flow rate of 0.6 ml/min, mobile phase constituted of deionized water: acetonitrile:ammonium acetate (10 Mm) (14.5:85.0:0.5; v/v/v), and the sample injected volume of 10 μL. The run was conducted at room temperature for a duration of 15 min.

### Estimate exposure calculation

Exposure to penicillin residues was computed using the tiered assessment approach suggested by JECFA [32]. The maximum dietary exposure to penicillin residues from beef consumption from the different parts of meat (muscle, liver, and kidney) was calculated in Tier 1. The mean dietary exposure to penicillin residues from the different parts of meat (muscle, liver, and kidney) was calculated in Tier 2. The total dietary exposure to penicillin residues was calculated in Tier 3 using the following equations:

Tier 1: Maximum dietary exposure to penicillin (µg/day/person)=∑Maximum permitted usage of penicillin (µg/kg)×maximum meat consumption (kg/day/person).
Tier 2: Mean dietary exposure to penicillin (µg/day/person)=∑Maximum permitted usage of penicillin (µg/kg)×mean meat consumption (kg/day/person).
Tier 3: Dietary exposure to penicillin (µg/person/day)=ΣMedian consumption value of beef meat (g/person/day)×average penicillin residue level (µg/g).

The mean dietary exposure to penicillin residues in selected sociodemographic groups that had a markedly higher consumption of meat such as males, obese subjects, those with an income <999,000 Lebanese pound (LBP), and those with an educational level less than brevet was also calculated using the same equation.

### Statistical analyses

The Statistical Package for the Social Sciences (SPSS) version 22 (IBM, USA) for Windows was used for data entry and analyses. Univariate analyses were used to assess quantitative variables and qualitative variables. Frequencies and percentages were calculated for qualitative variables such as gender, level of education, marital status, area of residence, level of education, and monthly household income, while mean and standard deviation were calculated for quantitative variables such as total red meat consumption as well as specific red meat product consumption. Bivariate analyses were used to compare different groups and estimate associations regarding the effect of age, income, education, gender, and other sociodemographic characteristics on red meat and red meat items consumption in the Lebanese population. Furthermore, associations between antibiotic knowledge and antibiotic intake were assessed, and the effects of sociodemographic characteristics on antibiotic knowledge and on antibiotic intake were studied. Statistical methods used to analyze our data included independent t-test to compare the amount of consumption between two groups, one-way ANOVA followed by Duncan *post hoc* test was used to compare the means of other indices as well as Chi-square to test the relationship among qualitative variables. p<0.05 was considered statistically significant.

## Results

Five hundred participants were recruited in December 2016, with an average age of 40.4±13.3 years with 58.8% being female (Table-1). Of the participants, 53.4% had at least a Bachelor’s degree, 48.0% were married, and 66.2% had a monthly household income <2,999,000 LBP (equivalent to 1999.00 US $) which is 5 times more than minimum Lebanese salary (425.00 US $). Among the participants, 51.2% had a normal BMI (BMI 18.5-24.9 kg/m^2^), whereas 37.8% were overweight (BMI 25-29.9 kg/m^2^), 8.6% were obese (BMI >30 kg/m^2^), and 2.4% were underweight (BMI <18.5 kg/m^2^). Less than half of the participants (44%) reported using antibiotic on doctor’s prescription, 1.2% reported having allergies to red meat, and 7.8% reported having allergies to antibiotics.

**Table 1:** Sociodemographic characteristics of Lebanese consumers in Mount Lebanon.

Characteristics	n/mean±SD	%
Age, years	40.4±13.3	
<25	59	11.8
25-34	122	24.4
35-44	140	28.0
45-54	96	19.2
>54	83	16.6
Gender		
Male	206	41.2
Female	294	58.8
Highest level of education completed		
Less than brevet	37	7.4
Brevet	68	13.6
High school	128	25.6
BSc. degree	197	39.4
Master’s degree or higher	70	14.0
Current marital status		
Single	188	37.6
Married	240	48.0
Divorced	47	9.4
Widowed	25	5.0
Income (LBP^2^/month)		
<999,000	65	13.0
1,000,000-1,999,000	162	32.4
2,000,000-2,999,000	104	20.8
3,000,000-3,999,000	80	16.0
4,000,000-4,999,000	25	5.0
>5,000,000	64	12.8
BMI^1^ kg/m^2^		
Underweight (<18.5)	12	2.4
Normal (18.5-24.9)	256	51.2
Overweight (25-29.9)	189	37.8
Obese (>30)	43	8.6
Use of antibiotic with prescription		
Yes	220	44.0
No	280	56.0
Allergies on red meat consumption		
Yes	6	1.2
No	494	98.8
Allergies on antibiotic consumption		
Yes	39	7.8
No	461	92.2

1 BMI=Body mass index defined as the body mass divided by the square of the body height,

2 LBP=Lebanese pound with a rate of exchange of 1500 LBP=1.00 US$

Regarding their antibiotic use (Table-2), 66.4% reported that antibiotics are safe, 29.4% did not consult a doctor before starting an antibiotic, and 32.4% stopped it after taking few doses and starting to feel better. More than half of the participants knew that inappropriate use of antibiotics can lead to ineffective treatment (61.2%), worsen or increase the duration of illness (57.8%), lead to bacterial resistance (57.6%), and that if taken too often, they are less likely to work in the future (51.6%).

**Table 2:** The frequencies and answers on questions related to antibiotic knowledge of Lebanese consumers in Mount Lebanon.

Statement (Correct answer)	Yes (%)	No
Antibiotics are safe drugs (No)	332 (66.4)	168 (33.3)
Do you consult a doctor before starting an antibiotic (Yes)	353 (70.6)	147 (29.4)
After taking 2-3 doses you start feeling better. Do you stop taking the further treatment? (No)	162 (32.4)	338 (67.6)
Can inappropriate use of antibiotics lead to ineffective treatment? (Yes)	306 (61.2)	194 (38.8)
Can inappropriate use of antibiotics worsen or increase the duration of illness? (Yes)	289 (57.8)	211 (42.4)
Can inappropriate use of antibiotics lead to bacterial resistance? (Yes)	288 (57.6)	212 (42.4)
If taken too often, antibiotics are less likely to work in the future. (Yes)	258 (51.6)	242 (48.4)

Overall, 62.8% of the study participants had moderate or poor antibiotic knowledge. A higher knowledge score about antibiotics was reported by females (p=0.018), belonging to the younger age group (18-35 years, p=0.024), with at least a Bachelor’s degree (p=0.000), a monthly income ≥5,000,000 LBP, and those who reported having allergies on consumption of antibiotics (p=0.013) (Table-3).

**Table 3:** Antibiotic knowledge score of Lebanese consumers in Mount Lebanon based on sociodemographic characteristics*.

Sociodemographic characteristic	n	Antibiotic knowledge score^1^ n (%)			p-value

		Poor	Moderate	Good	
Total population	400	173 (34.6)	141 (28.2)	186 (37.2)	
Gender					
Male	206	84 (40.8)	59 (28.6)	63 (30.6)	0.018
Female	294	89 (30.8)	82 (27.9)	123 (41.8)
Age, years					
<25	59	15 (25.4)	17 (28.8)	27 (45.8)	0.024
25-34	122	28 (23.0)	41 (33.6)	53 (43.4)	
35-44	140	53 (37.9)	35 (25.0)	52 (37.1)	
45-54	96	39 (40.6)	26 (27.1)	31 (32.3)	
>54	83	38 (45.8)	22 (26.5)	23 (27.7)
Education					
<Brevet	37	26 (70.3)	8 (21.6)	3 (8.1)	0.000
Brevet	68	41 (60.3)	19 (27.9)	8 (11.8)	
High school	128	57 (44.5)	37 (28.9)	34 (26.6)	
Bachelor’s degree	197	36 (18.3)	56 (28.4)	105 (53.3)	
Master’s degree and beyond	70	13 (18.6)	21 (30)	36 (51.4)
Current monthly income in LBP^2^					
<999,000	65	35 (53.8)	22 (33.8)	8 (12.3)	0.000
1,000,000-1,999,000	162	77 (47.5)	49 (30.2)	36 (22.2)	
2,000,000-2,999,000	104	24 (23.1)	29 (27.9)	51 (49.0)	
3,000,000-3,999,000	80	15 (18.8)	19 (23.8)	46 (57.5)	
4,000,000-4,999,000	25	11 (44.0)	7 (28.0)	7 (28.0)	
≥5,000,000	64	11 (17.2)	15 (23.4)	38 (59.4)
Marital status					
Single	188	63 (33.5)	52 (27.7)	73 (28.8)	0.960
Married	240	83 (34.6)	69 (28.8)	88 (36.7)	
Divorced	47	16 (34.0)	13 (27.7)	18 (38.3)	
Widowed	25	11 (44.0)	7 (28.0)	7 (28.0)
Allergies on consumption of antibiotics					
Yes	39	8 (20.5)	8 (20.5)	23 (59.0)	0.013
No	461	165 (35.8)	133 (28.9)	163 (35.4)	

1 =A poor score of 0-2/7, moderate score of 3-5/7, and good score of 6-7/7.

2 LBP=Lebanese pound.

* Chi-square was used to compare antibiotic knowledge among the different sociodemographic groups

**Figure-1:**
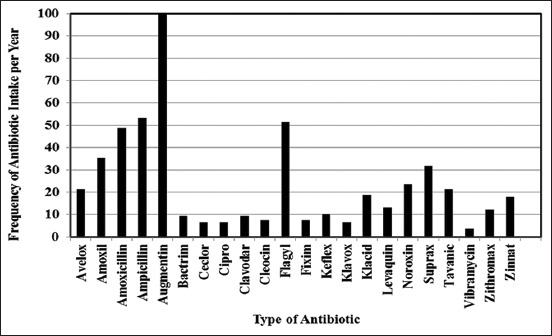
Frequency of antibiotic intake of Lebanese consumers in Mount Lebanon over 1 year in 2016.

**Figure-2:**
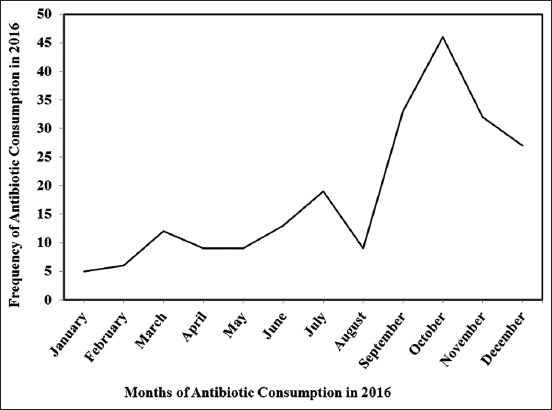
Frequency of antibiotic consumption over the months of 2016 among Lebanese consumers in Mount Lebanon.

Augmentin^™^ (100%) was the most frequently used antibiotic, followed by Ampicillin^™^ (53.2%), amoxicillin (48.6%), Flagyl (51.4%), and Amoxil (48.5%) (Figure-1). Almost half (47%) of the Lebanese consumed antibiotic in October and more than 30% would consume it between September and December (Figure-2).

Subjects who obtained a good score (6-7) on antibiotic knowledge had at least high school degree, and a minimum household monthly income of 2,000,000 LBP is significantly (p<0.05) more likely to have better antibiotic practices with lesser change of antibiotic during treatment and lower use of antibiotic as compared to those with poor antibiotic knowledge, lower education, and income (p>0.05) (Table-4).

**Table 4:** Attitudes and behaviors toward antibiotic intake based on sociodemographic characteristics of Lebanese consumers in Mount Lebanon.

Attitude/Behavior	n	Response (%)									

		Knowledge score				Education level			Monthly household income (LBP)		
		
		Poor n=173	Moderate n=141	Good n=186	p-value	Grade 9 or less n=105	High school and beyond n=395	p-value	<1,999,000 n=227	>2,000,000 \n=273	p-value
Change of antibiotic during treatment	58	48.6	55.3	31.2	0.000	10.5	11.9	0.210	13.2	10.3	0.259
Use antibiotics when having											
Fever (temperature<38.5°C)	114	32.4	27.0	10.8	0.000	52.4	14.9	0.000	36.1	11.7	0.000
Common cold	134	48.0	25.5	8.1	0.000	59.0	18.2	0.000	42.7	13.6	0.000
Acute bronchitis	335	63.6	70.9	67.2	0.387	68.6	66.6	0.700	65.2	68.5	0.435
Coughing up sputum	271	66.5	58.9	39.2	0.000	65.7	51.1	0.008	60.8	48.7	0.007
Sore throat	277	65.3	59.6	43.0	0.000	71.4	51.1	0.000	64.8	47.6	0.000
Congested nose with headache	247	69.4	52.5	28.5	0.000	72.4	43.3	0.000	59.5	41.0	0.000
Coughing for more than 2 weeks	325	72.3	67.4	56.5	0.006	74.3	62.5	0.025	69.2	61.5	0.075

LBP=Lebanese pound

Participants reported consuming mostly cow meat as compared to lamb and goat meats, the daily intakes were 191.3 g/day, 23.2 g/day, and 3.3 g/day, respectively (Table-5). Ground meat and muscle meat were mostly consumed from beef with a mean of 113.7 g/day and 47.3 g/day, respectively, as compared to organ meat, raw meat, and sausages with mean daily intake of 6.5, 6.2, and 8.6 g/day, respectively. The average total beef intake was significantly higher among male (239.5±131.2 g/day, p=0.000), single subjects (214.0±136.4 g/day) (p=0.016), obese subjects (BMI >30 kg/m^2^) (241.1±155.7 g/day, p=0.043), and those with a monthly household income lower than 999,000 LBP (235.8±110.3 g/day, p=0.000) (Table-6).

**Table 5:** Meat consumption of Lebanese consumers in Mount Lebanon of beef, lamb, and goat per meat category.

Meat category	Beef	Lamb	Goat
		
	Consumption (g/day) [range]	Consumption (g/day) [range]	Consumption (g/day) [range]
Minced meat	113.7±72.7 [0-331.7]	6.6±2.1 [0-154.7]	0.9±6.6 [0-74.1]
Organ meat	6.5±10.3 [0-38.5]	3.5±1.2 [0-81.8]	0.2±3.0 [0-53.3]
Raw meat	6.2±10.6 [0-71.3]	4.6±1.4 [0-103.3]	0.3±2.9 [0-57.0]
Muscle meat	47.3±35.2 [0-165.7]	3.6±1.0 [0-64.4]	0.4±3.4 [0-42.8]
Sausages	8.6±9.6 [0-35.2]	0.09±1.0 [0-16.6]	0.007±0.15 [0-3.3]
Total	191.3±1.2 [0-530.0]	23.2±5.6 [0-290.4]	3.3±1.4 [0-108.9]

Consumption values are mean±standard deviation [min, max range]

**Table 6:** Beef consumption values based on sociodemographic characteristics of Lebanese in Mount Lebanon.

Sociodemographic characteristic	Total beef consumption (g/day)	p-value
Gender		
Male	239.5±131.2	0.000
Female	158.8±103.2	
Age (years)		
<25	204.9±139.5	0.323
25-34	190.4±125.9	
35-44	192.7±117.2	
45-54	192.7±117.2	
>54	171.4±114.7	
Marital status		
Single	214.0±136.4	0.016
Married	176.9±110.4	
Divorced	182.2±110.7	
Widowed	174.5±107.6	
BMI		
Underweight	175.9±100.5	0.043
Normal	181.8±118.1	
Overweight	195.5±117.7	
Obese	241.1±155.7	
Highest level of education		
Less than brevet	229.4±99.7	0.106
Brevet	215.8±113.2	
High school	183.3±119.6	
BSc. degree	187.0±134.2	
Master’s degree and beyond	176.4±101.8	
Income (LBP/month)		
<999,000	235.8±110.3	0.000
1,000,000-1,999,000	198.5±125.2	
2,000,000-2,999,000	185.4±138.1	
3,000,000-3,999,000	193.4±128.5	
4,000,000-4,999,000	199.7±88.2	
>5,000,000	131.2±81.5	

Consumption values are mean±standard deviation (95% confidence interval); ANOVA test was used to compare mean among different sociodemographic groups. LBP=Lebanese pound

Among the assessed meat samples (n=61), 21.3% contained penicillin above the LOD, making the consumers exposed to penicillin residues once every 5 times they eat meat (Table-7). The detected penicillin residue levels were all below the MRL set at 50 μg/kg (MRL) by Codex Alimentarius – International Food Standard MRLs [33] and ranged between a minimum of 5.1 and a maximum of 8.9 μg/kg with an average residue of 6.1±1.5 μg/kg. The highest residues were detected in steak meat (6.6±1.2 μg/kg), followed by minced meat (6.2±1.7 μg/kg), liver meat (6.1±1.3 μg/kg), and the lowest in kidney meat (3.2±0 μg/kg).

**Table 7:** Detected level of penicillin residues in beef samples.

Beef product	Positive^a^/n	Positive (%)	Minimum (μg/kg)	Maximum (μg/kg)	Mean±Standard deviation (μg/kg)
Total	13/61	21.3	5.1	8.9	6.2±1.2
Steak	5/25	20.0	5.6	8.2	5.9±1.3
Minced beef	5/23	21.7	5.2	8.9	6.8±1.3
Liver	2/9	22.2	5.1	5.4	5.2±0.2
Kidney	1/4	25.0	5.1	5.2	5.2±0

a Positive samples: samples in which the antibiotic residue levels exceeded the limits of detection (LOD). LOD of the RIDASCREEN^®^ Penicillin ELISA kit is 5.00 μg/kg

Using the tiered approach, the daily intake of penicillin residues through long-term meat consumption by the Lebanese was estimated to be 26.5, 9.6, and 1.7 µg/kg bw/day using Tier 1, 2, and 3, respectively (Table-8). The results also showed that 88.3% of the acceptable daily intake (ADI) was achieved when using Tier 1. All of the male, single, obese participants, and those with household monthly income below 999,000 LBP were reaching between 35.7 and 40.2% of the ADI of penicillin established by JECFA [32] (30 µg/day/person).

**Table 8:** Exposure assessment of the Lebanese consumers to penicillin through consumption of beef.

Variables	Meat consumption (kg)	Residue level (μg penicillin/kg)	EDI (μg/kg bw/day)	%ADI
Tier 1	0.530	50	26.5	88.3
Tier 2	0.191	50	9.6	31.9
Tier 3	0.191	8.9	1.7	5.7
Male	0.240	50	12.0	39.9
Single	0.214	50	10.7	35.7
Obese	0.241	50	12.1	40.2
Household monthly income<999,000	0.236	50	11.8	39.3

EDI=Effective drought index, ADI=Acceptable daily intake

## Discussion

### Exposure to penicillin through medication intake

This study reveals that most of the Lebanese participants have moderate to poor knowledge of antibiotic medications. In addition, those with poor antibiotic knowledge, lower education, and income are more likely to change antibiotic during treatment and improperly use it.

More than half of the participants knew that antibiotic misuse leads to ineffective treatment and bacterial antimicrobial resistance, yet more than half would use it without prescription. Jamhour *et al*. [25] reported similar rates of self-medication (51%) among 400 participants living in Beirut city and Tripoli. Similarly, high rates were reported in Abu Dhabi (46%) [34] and Kuwait (63.1%) [21]. This rate was much higher than those previously reported by Mouhieddine *et al*. [24] and Cheaito *et al*. [35] among Lebanese consumers living in Beirut (23% and 42%, respectively) and by Yusef *et al.*, Shehadeh *et al.*, and Sawair *et al*. [36-38] among Jordanian participants (38.0%, 30.0%, and 40.7%, respectively). The prevalence of self-medication with antibiotics reported in the previous studies was higher than that reported among 28 European countries ranging between 2% in Sweden and 21% in Greece (Special Eurobarometer 445 summary antimicrobial resistance, 2016). It is worth highlighting that the use of antibiotics without prescription seems to increase over the years, and this could be justified by the ease of access to antibiotics due to the lack of regulation concerning antibiotic dispensation and the inability of many people to afford a medical visit [23]. This highlights the need to reinforce the law and control the pharmacist practices [39].

The present study indicates that 62.8% of the study participants had moderate or poor score on antibiotic knowledge. In fact, about half of the participants knew that if antibiotic is taken too often, they are less likely to work in the future and that inappropriate use of antibiotics could lead to ineffective treatment, bacterial resistance, or adverse effects. Similarly, Mouhieddine *et al*. [24] reported that 62.4% of the participants had poor to moderate antibiotic knowledge. These authors also highlighted that approximately 53.1% of the participants know that they should complete the full course of antibiotics even when their symptoms improve and 51.5% said that they would stop taking their course of antibiotics if their symptoms disappear. Khan and Banu [40] reported that among medical students (n=97) who were more knowledgeable of health, a higher percentage (92.8%) would always consult a doctor and 74.2% would complete the full course treatment. The high percentage of participants in our study who are not aware that antibiotic misuse has several side effects may imply that patients are not getting adequate information from health-care professionals regarding the medications they use [24]. The prevalent poor knowledge of the subjects can be due to a lack of proper medical awareness in the Lebanese population. This poor knowledge could be alarming as it may significantly contribute to the increase in resistant bacterial infections in Lebanon. This highlights the immediate need for policies on antibiotic prescription and raising awareness of antibiotic use among the Lebanese population.

In agreement with the study findings, several studies highlighted that participants with lower antibiotic knowledge were men, belonging to an older age group, with low education and low monthly income [24,41,42]. Mouhieddine *et al*. [24] reported that among 495 Lebanese participants living in Beirut, the level of knowledge was significantly associated with monthly income, place of residence, educational level, spending a year abroad, working in the medical sector, or having a relative who works in the medical sector, in addition to having insurance, especially one that covers medication costs. Jamhour *et al*. [25] also reported that among 400 participants, a significant association was common between low educational level, knowledge, and the misuse of antibiotics in Beirut and Tripoli; however, the impact of economic status did not show to be statistically significant. Similarly, other studies like Vallin *et al*. [42] reported that among Swedish adult men (n=2500), higher education and younger age group were associated with better knowledge of antibiotics and Napolitano *et al*. [41] reported that among parents (n=419) in Italy, participants with a higher education were more knowledgeable of antibiotic resistance. According to our findings, awareness among the Lebanese community on antibiotic usage should be reinforced where those with low sociodemographic characteristics would be the main target and where pharmacists and doctors in Lebanon would have an important role in teaching and advising.

In accordance with our findings, the previous studies in Lebanon [25], Malaysia [43], and South Korea [44] showed that poor sociodemographic characteristics specifically low education, low income, and low antibiotic knowledge were reported as factors that are significantly associated with poor attitude of antibiotic usage. In addition, higher knowledge of antibiotics was associated with a more “appropriate restrictive” attitude toward antibiotics [21,24,43,44,]. On the other hand, Farah *et al*. [23] reported that pharmacies located in lower socioeconomic areas are more likely to dispense antibiotics without prescription as compared to those in higher socioeconomic areas and this could be due to the inability of many people to afford a medical visit. Those results highlight that improving people’s knowledge would enable them to make correct decisions when taking antibiotics with respect to time, dosage, and appropriateness of treatment [44]. Those results also highlight the need to raise awareness among the Lebanese in general and those with lower education and income more specifically.

In this study, penicillin-containing antibiotics were among the most used in Mount Lebanon over 1 year in 2016. Similarly, penicillin antibiotics were also the most commonly consumed antibiotics in Europe [45] and Abu Dhabi where amoxicillin and augmentin were the most used [34]. Cheaito *et al*. [35] reported that penicillin-containing antibiotic amoxicillin was the most frequently taken without prescription. Other studies conducted in Sudan, Jordan, India, and the United Arab Emirates also reported that amoxicillin was the most commonly consumed antibiotic without prescription [34,46-48]. Penicillin antibiotics are among the most consumed antibiotics since they treat a wide range of bacteria including common infections in the ear, throat, skin, scarlet fever, and pneumonia [49]. In addition, among those who reported allergies on consumption of antibiotics, 45.4% took antibiotics containing penicillin in the course of 2016. The high intake of penicillin antibiotics that were reported among participants could possibly contribute to penicillin resistance and increase in allergic reaction in Lebanon.

The antibiotic use increased in the cold season between September and December from 30 and 47%. In line with our study, Suda *et al*. [50] also reported that in the United States, antibiotic prescriptions were 24.5% higher in winter months than in the summer, and 75% and 100% increase in penicillin and macrolide prescriptions were reported during winter months. This seasonal variation was also reported in Europe [51] and in the United States [52]. A significant seasonal variation could be mainly related to the increase in the incidence of respiratory tract infections [50]. It was thus suggested that efforts to reduce inappropriate antibiotic use may have the most impact if initiated during or just before the cold season.

### Exposure to penicillin through residues in meat

The present study reported high total daily consumption of red meat from beef (191.3 g/day). Similarly, red meat intakes were reported in Sweden (190.9 g/day), Spain (194.2 g/day), Germany (204.9 g/day), and the Netherlands (215 g/day) [53]. However, it was much higher than that reported previously among participants (n=444) living in Beirut with a mean value of 47.6 g/day [54] and 76 g/day and 51 g/day in Iran [55,56]. Beef consumption was lower in Australia (173 g/day) [19], Canada (156 g/day) [53], Ireland (152.6 g/day) [57], Italy (151.7 g/day) [53], the United States (128 g/day) [58], and Croatia (126 g/day) [59]. Moreover, beef consumption (191.3 g/day) was higher than the recommendation of the World Cancer Research Fund and UK guidelines, which is to limit meat intake to <70 g/day and the United States Department of Agriculture recommendation of lean red meat intake of 48 g/day. Meat consumption in the Middle East was reported to be on the rise since 2007 for the upcoming years due to rapid industrialization [38]. Furthermore, Zaki *et al*. [27] reported that bovine meat consumption is on the rise in Mount Lebanon regions. This increase in meat consumption in the Middle East and particularly Lebanon further explains the high beef consumption values reported in our study. This high meat intake could be associated with various health risks and result with high exposure to antibiotic and other veterinary drug residues.

The results show that the average total beef consumption was significantly the highest among men, single subjects, obese, and those with an income <999,000 LBP. In line with our study, Falahi *et al*. [55] reported that consumers aged <30 years consume more beef than older ones. In Florida, the younger age group and those having a lower level of education had higher beef consumption than the other groups [60]. In Portugal, lower income and lower education groups also had higher beef consumption than the rest of the population [61]. In contrast to our results, the Jordan’s Window Global Competitiveness in 2007 related increased incomes with the rise in meat consumption in the Middle East. In Florida also, higher income levels were associated with increased beef consumption since it is more expensive than other types of meat such as chicken, pork, and fish [60]. Although some studies attributed higher incomes with an increase in beef consumption, the decrease in beef consumption reported in our study in higher income and higher education groups can be due to the fact that these groups tend to be more health conscious or more likely to have healthier lifestyles [62] and tend to experiment more with different food options [60].

Higher consumption of beef was seen in subjects with high BMI, which can be due to the fact that overweight and obese subjects that are otherwise healthy tend to eat more energy-dense food containing high amounts of fat and less healthy alternatives such as lean poultry, fish, and legumes [63]. Therefore, strategies aiming to reduce red meat consumption should primarily those groups in the population.

The prevalence of penicillin residues, of this study, was 21.3% in the selected beef samples, making the consumers exposed to antibiotic once every 5 times they consume meat. In Marowa, Cameron, the prevalence of penicillin G, in cattle liver (n=202) and muscle (n=202) samples was 18.8%, of which 28.95% had a concentration higher than the MRL [5]. In Nigeria, among beef liver (n=50) and kidneys (n=50), 44% of the total samples were positive for penicillin residues. The prevalence of penicillin in the positive liver (n=32) and kidney (n=12) samples was 14% [64]. In Gaza, the prevalence of penicillin in chicken muscle meat was 21% [65]. The prevalence of total antibiotic residues in beef in Nigeria was 54.4%, Kenya 45.6%, Ghana 30.8%, and Sudan 17.33% [6]. In 2003-2005, the Food and Drug Administration issued a total of 344 warning letters to cattle producers, of which 34% targeted penicillin residues violation [66]. Several factors affect the high prevalence of antibiotic residues in meat, especially in developing countries. The main reasons include (1) lack of knowledge regarding withdrawal periods among both farmers and butchers; (2) lack of communication with the veterinary consultants; (3) lack of monitoring and surveillance of antibiotic administration; (4) inappropriate and even absence of testing and validation of safety for antibiotic residues; and (5) absence of certification of the imported beef products [7,67]. In this study, minced meat showed the greatest incidence of penicillin residues, followed by steak, liver, and the least was kidney. Similarly, Geidman *et al*. [68] reported that muscle meats have a greater incidence of residues (15.7%) of penicillin as compared to 13% in liver and 8.3% in kidneys. In Sudan, the highest prevalence of penicillin was also detected in muscles (29.3%) followed by liver (28.3%) and the least in kidney (21.4%) [36]. The highest concentrations of penicillin residues were also detected in steak meat and the lowest in the kidney sample. In line with those results, Adesokan *et al*. [69] detected penicillin residues in muscle meat, liver, and kidneys to be 11.7±2.9, 8.5±2.8, and 6.3±2.5 µg/kg, respectively, with no significant differences in mean residue levels (F=29.4, df=2.9) in Nigeria. Penicillin residues ranging from 0.07 to 81.45 µg/kg were detected in 675 cases in cattle products worldwide [70]. In Cameroon, the mean penicillin G concentration (n=404) was 17.58 µg/kg, with a range from 0 to 1 mg/kg which is 20 times higher than the MRL, where the liver samples (n=202) contained higher median concentration of penicillin (0.087 µg/kg) than muscles (n=202) (0.055 µg/kg) [5]. This could be due to the fact that the point of injection of antibiotics in cattle is intramuscular and in the neck region, and all excess residues are stored in muscles and fat cells of the cattle [71,72]. Moreover, the elimination of antibiotics occurs at different levels in cattle primarily in the liver and kidneys, leading to lower concentrations of penicillin in those organs as compared to the muscles. However, the toxicity of the metabolites is of concern in this case [72].

The estimated exposure assessment to penicillin residues from the dietary intake of red meat is high if we take into consideration the maximum intake, acceptable if considering the average meat intake, and low if considering the total dietary intake. The results also show if assessing the effective drought index of sociodemographic groups with the highest consumption of meat they are achieving almost 40% of the ADI. Although the risk is considered negligible for most of the population, those with high meat intake are highly exposed. Moreover, it is suggested that penicillin residue concentration as low as 6 µg/kg and a total dietary intake of 0.1 µg/person/day are enough to induce an anaphylactic reaction in humans, taking into consideration a standard weight as reported by the European Committee for Veterinary Medicinal Products [73]. Unintentional consumption of penicillin residues can lead to many health effects, in addition to allergic reactions, neurotoxicity, severe inflammation of the colon, swelling of the lips, face and tongue, bleeding, and diarrhea [70]. Although 1.2% of the population had allergies on the consumption of red meat, it is difficult to link the allergies to antibiotic residues found in red meat, especially penicillin which is known to cause allergies in humans. Penicillin-sensitive individuals are at the greatest risk of penicillin-induced anaphylactic reactions and other cutaneous symptoms with exposure to minute concentrations in meat as low as 0.02 µg/kg [74,75]. It has been postulated that chronic toxicity occasionally occurs from exposure to minute amounts of some residues resulting in bioaccumulation and transformation into carcinogens over prolonged periods of time [76]. Hence, Lebanese consumers might not be at risk of exposure to penicillin residues from meat intake but could be at risk of anaphylactic reactions and bioaccumulation of residues and their transformation to carcinogens overtime. This suggests the need for enforcing food safety laws and regulations, incriminating pharmacies giving antibiotics without any prescription or allowing over-the-counter purchase of antibiotics with no valid reason, enforcing doctors to administer a bacteriological test before prescribing antibiotics to target the specific bacteria, educating farmers about proper antibiotic handling, monitoring and only using antibiotics when needed on the supervision of a veterinary, and finally launching awareness campaigns targeting all the public at different levels.

### Strengths and limitations of the study

Food safety, including application of antibiotics, is an issue of prime concern in Lebanon [77]. Our study is the first of its kind in the country. However, this type of study is commonly associated with some limitations in terms of bias in falsely reporting good practices. The FFQ is a commonly used tool to assess food intake at a low cost, minimal time, and trouble to the participants; however, participants could have problems in estimating portion sizes which could lead to over- or under-estimation of the red meat intake [78].

## Conclusion

The overall results suggested that the Lebanese with poor antibiotic knowledge, low education and income overuse, and misuse antibiotics, especially penicillin. Lebanese consumers are also exposed to penicillin residues every 5^th^ time they consume meat; however, the total dietary intake is below the ADI. Men, single, obese, and those participants with low income are the most highly exposed. Although the daily intake is low, consumers could be at risk of anaphylactic reactions and bioaccumulation of residues and their transformation to carcinogens. Several interventions could be implemented in Lebanon to reduce antibiotic resistance and sustain their effectiveness, such as adopting the Global Action Plan on Antimicrobial Resistance (Global Antibiotic Resistance Partnership), which includes improving water, hygiene, and vaccination, controlling infections in hospitals, reducing antibiotic use in humans and in agriculture, increasing consumer’s and health professionals’ awareness regarding the use of antibiotics, and developing policies on that matter. Finally, our study can be a benchmark for future studies on red meat consumption in other regions in Lebanon and in rural areas as well. It can also open prospective research in Lebanon on the exposure assessment to other antibiotic residues in red meat as well as residues in milk products, crops, and water.

## Authors’ Contributions

PHB and JM: Collected and analyzed data and drafted the manuscript. HH and CB: Conceptualized and designed the study. NE: Carried out the statistical analysis. EBY: Facilitated the sample collection. HH, MAJ, and CBM: Reviewed the final draft. JEF: Analyzed data and reviewed the final draft. All authors read and approved the final manuscript.
